# From Fear to Confidence: A Qualitative Study of Intergenerational Technology Support Among Low-Income Older Adults

**DOI:** 10.1093/geroni/igaf122.4126

**Published:** 2025-12-31

**Authors:** Andrew Kim, John Kim, Carissa Liu, Ian Lee, Mengzhao Yan, Min-Kyoung Rhee

**Affiliations:** University of Southern California, Los Angeles, California, United States; University of Southern California, Los Angeles, California, United States; University of Southern California, Los Angeles, California, United States; University of California, Los Angeles, Los Angeles, California, United States; University of Southern California, Los Angeles, California, United States; University of Southern California, Los Angeles, California, United States

## Abstract

Despite the growing reliance on technology for daily tasks, health management, and social connection, older adults, particularly those of advanced age and low income, continue to have lower rates of technology adoption. However, research shows that when factors like device access and learning opportunities are present, age and income are no longer significant barriers. Training and support also improve confidence in technology use among older adults. This study conducted a qualitative investigation into how intergenerational technical support can enhance technology use among low-income older adults. Participants aged 60 and older, living in affordable senior housing in Los Angeles, received weekly one-hour, one-on-one sessions with university student “tech buddies” over five weeks. They learned to use the devices and applications they were most interested in. Twenty-five participants who completed at least three sessions were included in the final sample, and semi-structured interviews were conducted post-intervention. Using grounded theory and constant comparison methods, we identified five major barriers to technology use: limited infrastructure, restricted access to support, age-related physical and cognitive changes, lack of knowledge and experience, and emotional resistance. Intergenerational support helped mitigate these challenges and led to seven positive outcomes, organized into four main themes: gratitude and satisfaction, improved tech knowledge and skills, interpersonal connection, and empowerment. Findings underscore the potential of personalized, intergenerational support to bridge the digital divide among low-income older adults. The study offers insights for community organizations and local government aiming to promote digital inclusion through targeted support and training for this underserved population.

